# Supplementary material for: External morphometric and microscopic analysis of the reproductive system in in- vitro reared stingless bee queens, Heterotrigona itama, and their mating frequency
Source: PLoS One. 2024 Sep 24;19(9):e0306085. doi: 10.1371/journal.pone.0306085 (PMC11421791; doi:10.1371/journal.pone.0306085)
Supplement: S1 Table — (DOCX) [file pone.0306085.s001.docx]

**Table S1** Microsatellite loci used were derived from *Trigona carbonaria*. Flanking primers, optimal annealing temperatures (Ta), expected number of allele and allele size for PCR are shown.

| **Locus** | **Repeat**  **motif** | **Primer sequence (5′ → 3′)** | **Ta (°C)** | **Alleles size range (bp)** | **Number of alleles per locus** | **Reference** |
| --- | --- | --- | --- | --- | --- | --- |
| TC3.155 | (TC)_12_ | F: AGAATCACGTCGGCATCCGGA | 58 | 153 – 165 | 5 | Green et al. (2001) |
|  |  | R: CTTGAAATCCAGCGCAGAGTG |  |  |  |  |
| TC4.287 | (GGA)_9_ | F: TCCACCGCGATACGATGGTAC | 58 | 180 – 184 | 6 | Green et al. (2001) |
|  |  | R: GTAATACAACGCGGCTTCCTC |  |  |  |  |
| TC7.13 | (CAA)_12_ | F: GTAACGTGCCACCAGCTTTCG | 58 | 146 – 150 | 4 | Green et al. (2001) |
|  |  | R: GAGCGATCAAAGTGACCAGTC |  |  |  |  |
