# Supplementary material for: External morphometric and microscopic analysis of the reproductive system in in- vitro reared stingless bee queens, Heterotrigona itama, and their mating frequency
Source: PLoS One. 2024 Sep 24;19(9):e0306085. doi: 10.1371/journal.pone.0306085 (PMC11421791; doi:10.1371/journal.pone.0306085)
Supplement: S2 Table — (DOCX) [file pone.0306085.s002.docx]

**Table S2** Morphometric characters measurements were taken for in-vitro queens (n=11), natural virgin queens (n=22), and workers (n=30) of *Heterotrigona itama* from Thailand.

| **Morphometric characters** | **Case** | **Mean value** | **Std. Deviation** | **Std. Error** |
| --- | --- | --- | --- | --- |
| Head width (HW) | *In-vitro* queen | 2.25 | 0.05138 | 0.01549 |
|  | natural queen | 2.2636 | 0.03374 | 0.00719 |
|  | worker | 2.39 | 0.01965 | 0.00359 |
| Head length (HL) | *In-vitro* queen | 2.0155 | 0.12283 | 0.03703 |
|  | natural queen | 2.165 | 0.06592 | 0.01405 |
|  | worker | 2.0403 | 0.02173 | 0.00397 |
| Compound eye width (CEW) | *In-vitro* queen | 0.2882 | 0.01163 | 0.00351 |
|  | natural queen | 0.2829 | 0.01025 | 0.00218 |
|  | worker | 0.3695 | 0.02348 | 0.00429 |
| Compound eye length (CEL) | *In-vitro* queen | 1.1918 | 0.00751 | 0.00226 |
|  | natural queen | 1.1891 | 0.01411 | 0.00301 |
|  | worker | 1.4643 | 0.04074 | 0.00744 |
| Mandible length (ML) | *In-vitro* queen | 0.8835 | 0.04525 | 0.01364 |
|  | natural queen | 0.8863 | 0.02317 | 0.00494 |
|  | worker | 0.8565 | 0.00971 | 0.00177 |
| Apex length (ApL) | *In-vitro* queen | 0.2858 | 0.00464 | 0.0014 |
|  | natural queen | 0.2895 | 0.0063 | 0.00134 |
|  | worker | 0.2538 | 0.00358 | 0.00065 |
| Head width (HW) | *In-vitro* queen | 2.25 | 0.05138 | 0.01549 |
|  | natural queen | 2.2636 | 0.03374 | 0.00719 |
|  | worker | 2.39 | 0.01965 | 0.00359 |
| Head length (HL) | *In-vitro* queen | 2.0155 | 0.12283 | 0.03703 |
|  | natural queen | 2.165 | 0.06592 | 0.01405 |
|  | worker | 2.0403 | 0.02173 | 0.00397 |
| Compound eye width (CEW) | *In-vitro* queen | 0.2882 | 0.01163 | 0.00351 |
|  | natural queen | 0.2829 | 0.01025 | 0.00218 |
|  | worker | 0.3695 | 0.02348 | 0.00429 |
| Compound eye length (CEL) | *In-vitro* queen | 1.1918 | 0.00751 | 0.00226 |
|  | natural queen | 1.1891 | 0.01411 | 0.00301 |
|  | worker | 1.4643 | 0.04074 | 0.00744 |
| Mandible length (ML) | *In-vitro* queen | 0.8835 | 0.04525 | 0.01364 |
|  | natural queen | 0.8863 | 0.02317 | 0.00494 |
|  | worker | 0.8565 | 0.00971 | 0.00177 |
| Apex length (ApL) | *In-vitro* queen | 0.2858 | 0.00464 | 0.0014 |
|  | natural queen | 0.2895 | 0.0063 | 0.00134 |
|  | worker | 0.2538 | 0.00358 | 0.00065 |
| Basitarsus width (BW) | *In-vitro* queen | 0.3579 | 0.00524 | 0.00158 |
|  | natural queen | 0.3563 | 0.00777 | 0.00166 |
|  | worker | 0.5655 | 0.00505 | 0.00092 |
| Basitarsus length (BL) | *In-vitro* queen | 0.9415 | 0.00266 | 0.0008 |
|  | natural queen | 0.9469 | 0.01002 | 0.00214 |
|  | worker | 1.2739 | 0.15214 | 0.02778 |
| Fore wing width (FWW) | *In-vitro* queen | 2.1782 | 0.02228 | 0.00672 |
|  | natural queen | 2.1664 | 0.02804 | 0.00598 |
|  | worker | 2.4441 | 0.06226 | 0.01137 |
| Fore wing length (FWL) | *In-vitro* queen | 5.0664 | 0.13223 | 0.03987 |
|  | natural queen | 5.0673 | 0.13285 | 0.02832 |
|  | worker | 5.7259 | 0.0702 | 0.01282 |
| Marginal cell length (MCL) | *In-vitro* queen | 1.6964 | 0.04884 | 0.01473 |
|  | natural queen | 1.7077 | 0.05529 | 0.01179 |
|  | worker | 2.198 | 0.00962 | 0.00176 |
| 1stsubmarginal cell length (SCL) | *In-vitro* queen | 0.4863 | 0.0093 | 0.0028 |
|  | natural queen | 0.4858 | 0.01059 | 0.00226 |
|  | worker | 0.5421 | 0.01854 | 0.00339 |
| Hind wing width (HWW) | *In-vitro* queen | 1.0882 | 0.03545 | 0.01069 |
|  | natural queen | 1.0755 | 0.02703 | 0.00576 |
|  | worker | 1.1303 | 0.01629 | 0.00297 |
| Morphometric characters | Case | Mean value | Std. Deviation | Std. Error |
| Basitarsus width (BW) | *In-vitro* queen | 0.3579 | 0.00524 | 0.00158 |
|  | natural queen | 0.3563 | 0.00777 | 0.00166 |
|  | worker | 0.5655 | 0.00505 | 0.00092 |
| Basitarsus length (BL) | *In-vitro* queen | 0.9415 | 0.00266 | 0.0008 |
|  | natural queen | 0.9469 | 0.01002 | 0.00214 |
|  | worker | 1.2739 | 0.15214 | 0.02778 |
| Fore wing width (FWW) | *In-vitro* queen | 2.1782 | 0.02228 | 0.00672 |
|  | natural queen | 2.1664 | 0.02804 | 0.00598 |
|  | worker | 2.4441 | 0.06226 | 0.01137 |
| Fore wing length (FWL) | *In-vitro* queen | 5.0664 | 0.13223 | 0.03987 |
|  | natural queen | 5.0673 | 0.13285 | 0.02832 |
|  | worker | 5.7259 | 0.0702 | 0.01282 |
| Marginal cell length (MCL) | *In-vitro* queen | 1.6964 | 0.04884 | 0.01473 |
|  | natural queen | 1.7077 | 0.05529 | 0.01179 |
|  | worker | 2.198 | 0.00962 | 0.00176 |
| 1stsubmarginal cell length (SCL) | *In-vitro* queen | 0.4863 | 0.0093 | 0.0028 |
|  | natural queen | 0.4858 | 0.01059 | 0.00226 |
|  | worker | 0.5421 | 0.01854 | 0.00339 |
| Hind wing width (HWW) | *In-vitro* queen | 1.0882 | 0.03545 | 0.01069 |
|  | natural queen | 1.0755 | 0.02703 | 0.00576 |
|  | worker | 1.1303 | 0.01629 | 0.00297 |
| Morphometric characters | Case | Mean value | Std. Deviation | Std. Error |
| Basitarsus width (BW) | *In-vitro* queen | 0.3579 | 0.00524 | 0.00158 |
|  | natural queen | 0.3563 | 0.00777 | 0.00166 |
|  | worker | 0.5655 | 0.00505 | 0.00092 |
| Basitarsus length (BL) | *In-vitro* queen | 0.9415 | 0.00266 | 0.0008 |
|  | natural queen | 0.9469 | 0.01002 | 0.00214 |
|  | worker | 1.2739 | 0.15214 | 0.02778 |
| Fore wing width (FWW) | *In-vitro* queen | 2.1782 | 0.02228 | 0.00672 |
|  | natural queen | 2.1664 | 0.02804 | 0.00598 |
|  | worker | 2.4441 | 0.06226 | 0.01137 |
| Fore wing length (FWL) | *In-vitro* queen | 5.0664 | 0.13223 | 0.03987 |
|  | natural queen | 5.0673 | 0.13285 | 0.02832 |
|  | worker | 5.7259 | 0.0702 | 0.01282 |
| Marginal cell length (MCL) | *In-vitro* queen | 1.6964 | 0.04884 | 0.01473 |
|  | natural queen | 1.7077 | 0.05529 | 0.01179 |
|  | worker | 2.198 | 0.00962 | 0.00176 |
| 1stsubmarginal cell length (SCL) | *In-vitro* queen | 0.4863 | 0.0093 | 0.0028 |
|  | natural queen | 0.4858 | 0.01059 | 0.00226 |
|  | worker | 0.5421 | 0.01854 | 0.00339 |
| Hind wing width (HWW) | *In-vitro* queen | 1.0882 | 0.03545 | 0.01069 |
|  | natural queen | 1.0755 | 0.02703 | 0.00576 |
|  | worker | 1.1303 | 0.01629 | 0.00297 |
