# Supplementary material for: External morphometric and microscopic analysis of the reproductive system in in- vitro reared stingless bee queens, Heterotrigona itama, and their mating frequency
Source: PLoS One. 2024 Sep 24;19(9):e0306085. doi: 10.1371/journal.pone.0306085 (PMC11421791; doi:10.1371/journal.pone.0306085)
Supplement: S3 Table — The character abbreviations correspond to the S2 Table and S1 Fig. The asterisks indicate statistically significant differences. (DOCX) [file pone.0306085.s003.docx]

**Table S3** Multiple comparisons of 34 morphometric characters were conducted among *in-vitro* queens, natural virgin queens, and workers of *Heterotrigona itama*. The character abbreviations correspond to Table S2 and Figure S1. The asterisks indicate significant differences.

| **Morphometric characters** | **(I) Case** | **(J) Case** | **Mean Difference (I-J)** | **Std. Error** | ***P*-values** |
| --- | --- | --- | --- | --- | --- |
| HW | *in-vitro* queen | natural queen | -0.01364 | 0.01182 | 0.486 |
|  |  | worker | -.14000(*) | 0.01129 | <0.001 |
|  | natural queen | *in-vitro* queen | 0.01364 | 0.01182 | 0.486 |
|  |  | worker | -.12636(*) | 0.00899 | <0.001 |
|  | worker | *in-vitro* queen | .14000(*) | 0.01129 | <0.001 |
|  |  | natural queen | .12636(*) | 0.00899 | <0.001 |
| HL | *in-vitro* queen | natural queen | -.14959(*) | 0.02411 | <0.001 |
|  |  | worker | -0.02488 | 0.02302 | 0.53 |
|  | natural queen | *in-vitro* queen | .14959(*) | 0.02411 | <0.001 |
|  |  | worker | .12471(*) | 0.01833 | <0.001 |
|  | worker | *in-vitro* queen | 0.02488 | 0.02302 | 0.53 |
|  |  | natural queen | -.12471(*) | 0.01833 | <0.001 |
| CEW | *in-vitro* queen | natural queen | 0.00532 | 0.00667 | 0.706 |
|  |  | worker | -.08135(*) | 0.00636 | <0.001 |
|  | natural queen | *in-vitro* queen | -0.00532 | 0.00667 | 0.706 |
|  |  | worker | -.08667(*) | 0.00507 | <0.001 |
|  | worker | *in-vitro* queen | .08135(*) | 0.00636 | <0.001 |
|  |  | natural queen | .08667(*) | 0.00507 | <0.001 |
| CEL | *in-vitro* queen | natural queen | 0.00273 | 0.01096 | 0.966 |
|  |  | worker | -.27252(*) | 0.01046 | <0.001 |
|  | natural queen | *in-vitro* queen | -0.00273 | 0.01096 | 0.966 |
|  |  | worker | -.27524(*) | 0.00833 | <0.001 |
|  | worker | *in-vitro* queen | .27252(*) | 0.01046 | <0.001 |
|  |  | natural queen | .27524(*) | 0.00833 | <0.001 |
| ML | *in-vitro* queen | natural queen | -0.00277 | 0.00885 | 0.947 |
|  |  | worker | .02701(*) | 0.00845 | 0.006 |
|  | natural queen | *in-vitro* queen | 0.00277 | 0.00885 | 0.947 |
|  |  | worker | .02978(*) | 0.00673 | <0.001 |
|  | worker | *in-vitro* queen | -.02701(*) | 0.00845 | 0.006 |
|  |  | natural queen | -.02978(*) | 0.00673 | <0.001 |
| ApL | *in-vitro* queen | natural queen | -0.00373 | 0.0018 | 0.104 |
|  |  | worker | .03198(*) | 0.00172 | <0.001 |
|  | natural queen | *in-vitro* queen | 0.00373 | 0.0018 | 0.104 |
|  |  | worker | .03571(*) | 0.00137 | <0.001 |
|  | worker | *in-vitro* queen | -.03198(*) | 0.00172 | <0.001 |
|  |  | natural queen | -.03571(*) | 0.00137 | <0.001 |
| AnL | *in-vitro* queen | natural queen | .09182(*) | 0.02775 | 0.004 |
|  |  | worker | .65722(*) | 0.02649 | <0.001 |
|  | natural queen | *in-vitro* queen | -.09182(*) | 0.02775 | 0.004 |
|  |  | worker | .56540(*) | 0.0211 | <0.001 |
|  | worker | *in-vitro* queen | -.65722(*) | 0.02649 | <0.001 |
|  |  | natural queen | -.56540(*) | 0.0211 | <0.001 |
| HFW | *in-vitro* queen | natural queen | -0.00132 | 0.0029 | 0.892 |
|  |  | worker | .01118(*) | 0.00277 | <0.001 |
|  | natural queen | *in-vitro* queen | 0.00132 | 0.0029 | 0.892 |
|  |  | worker | .01250(*) | 0.0022 | <0.001 |
|  | worker | *in-vitro* queen | -.01118(*) | 0.00277 | <0.001 |
|  |  | natural queen | -.01250(*) | 0.0022 | <0.001 |
| HFL | *in-vitro* queen | natural queen | -0.01818 | 0.01022 | 0.185 |
|  |  | worker | .23930(*) | 0.00975 | <0.001 |
|  | natural queen | *in-vitro* queen | 0.01818 | 0.01022 | 0.185 |
|  |  | worker | .25748(*) | 0.00777 | <0.001 |
|  | worker | *in-vitro* queen | -.23930(*) | 0.00975 | <0.001 |
|  |  | natural queen | -.25748(*) | 0.00777 | <0.001 |
| HTW | *in-vitro* queen | natural queen | -0.00605 | 0.00633 | 0.607 |
|  |  | worker | -.03230(*) | 0.00604 | <0.001 |
|  | natural queen | *in-vitro* queen | 0.00605 | 0.00633 | 0.607 |
|  |  | worker | -.02625(*) | 0.00481 | <0.001 |
|  | worker | *in-vitro* queen | .03230(*) | 0.00604 | <0.001 |
|  |  | natural queen | .02625(*) | 0.00481 | <0.001 |
| HTL | *in-vitro* queen | natural queen | -0.01132 | 0.01628 | 0.767 |
|  |  | worker | -.08461(*) | 0.01554 | <0.001 |
|  | natural queen | *in-vitro* queen | 0.01132 | 0.01628 | 0.767 |
|  |  | worker | -.07329(*) | 0.01237 | <0.001 |
|  | worker | *in-vitro* queen | .08461(*) | 0.01554 | <0.001 |
|  |  | natural queen | .07329(*) | 0.01237 | <0.001 |
| BW | *in-vitro* queen | natural queen | 0.00164 | 0.00228 | 0.754 |
|  |  | worker | -.20759(*) | 0.00217 | <0.001 |
|  | natural queen | *in-vitro* queen | -0.00164 | 0.00228 | 0.754 |
|  |  | worker | -.20923(*) | 0.00173 | <0.001 |
|  | worker | *in vitro queen* | .20759(*) | 0.00217 | <0.001 |
|  |  | natural queen | .20923(*) | 0.00173 | <0.001 |
| BL | *in vitro queen* | natural queen | -0.00545 | 0.03912 | 0.989 |
|  |  | worker | -.33245(*) | 0.03734 | <0.001 |
|  | natural queen | *in vitro queen* | 0.00545 | 0.03912 | 0.989 |
|  |  | worker | -.32699(*) | 0.02974 | <0.001 |
|  | worker | *in-vitro* queen | .33245(*) | 0.03734 | <0.001 |
|  |  | natural queen | .32699(*) | 0.02974 | <0.001 |
| FWW | *in-vitro* queen | natural queen | 0.01182 | 0.01744 | 0.777 |
|  |  | worker | -.26588(*) | 0.01665 | <0.001 |
|  | natural queen | *in-vitro* queen | -0.01182 | 0.01744 | 0.777 |
|  |  | worker | -.27770(*) | 0.01326 | <0.001 |
|  | worker | *in-vitro* queen | .26588(*) | 0.01665 | <0.001 |
|  |  | natural queen | .27770(*) | 0.01326 | <0.001 |
| FWL | *in-vitro* queen | natural queen | -0.00091 | 0.03955 | <0.001 |
|  |  | worker | -.65950(*) | 0.03776 | <0.001 |
|  | natural queen | *in-vitro* queen | 0.00091 | 0.03955 | 1 |
|  |  | worker | -.65859(*) | 0.03007 | <0.001 |
|  | worker | *in-vitro* queen | .65950(*) | 0.03776 | <0.001 |
|  |  | natural queen | .65859(*) | 0.03007 | <0.001 |
| MCL | *in-vitro* queen | natural queen | -0.01136 | 0.01436 | 0.71 |
|  |  | worker | -.50167(*) | 0.01371 | <0.001 |
|  | natural queen | *in-vitro* queen | 0.01136 | 0.01436 | 0.71 |
|  |  | worker | -.49031(*) | 0.01091 | <0.001 |
|  | worker | *in-vitro* queen | .50167(*) | 0.01371 | <0.001 |
|  |  | natural queen | .49031(*) | 0.01091 | <0.001 |
| SCL | in vitro queen | natural queen | 0.00045 | 0.00548 | 0.996 |
|  |  | worker | -.05583(*) | 0.00523 | <0.001 |
|  | natural queen | *in-vitro* queen | -0.00045 | 0.00548 | 0.996 |
|  |  | worker | -.05628(*) | 0.00416 | <0.001 |
|  | worker | *in-vitro* queen | .05583(*) | 0.00523 | <0.001 |
|  |  | natural queen | .05628(*) | 0.00416 | <0.001 |
| HWW | *in-vitro* queen | natural queen | 0.01273 | 0.009 | 0.34 |
|  |  | worker | -.04215(*) | 0.00859 | <0.001 |
|  | natural queen | *in-vitro* queen | -0.01273 | 0.009 | 0.34 |
|  |  | worker | -.05488(*) | 0.00684 | <0.001 |
|  | worker | *in-vitro* queen | .04215(*) | 0.00859 | <0.001 |
|  |  | natural queen | .05488(*) | 0.00684 | <0.001 |
| HWL | *in-vitro* queen | natural queen | -0.00909 | 0.03558 | 0.965 |
|  |  | worker | -.16961(*) | 0.03396 | <0.001 |
|  | natural queen | *in-vitro* queen | 0.00909 | 0.03558 | 0.965 |
|  |  | worker | -.16052(*) | 0.02704 | <0.001 |
|  | worker | *in-vitro* queen | .16961(*) | 0.03396 | <0.001 |
|  |  | natural queen | .16052(*) | 0.02704 | <0.001 |
| TW4 | *in-vitro* queen | natural queen | -.03500(*) | 0.0125 | 0.019 |
|  |  | worker | .68436(*) | 0.01193 | <0.001 |
|  | natural queen | *in-vitro* queen | .03500(*) | 0.0125 | 0.019 |
|  |  | worker | .71936(*) | 0.0095 | <0.001 |
|  | worker | *in-vitro* queen | -.68436(*) | 0.01193 | <0.001 |
|  |  | natural queen | -.71936(*) | 0.0095 | <0.001 |
| TL4 | *in-vitro* queen | natural queen | -.13482(*) | 0.02926 | <0.001 |
|  |  | worker | 1.38910(*) | 0.02793 | <0.001 |
|  | natural queen | *in-vitro* queen | .13482(*) | 0.02926 | <0.001 |
|  |  | worker | 1.52392(*) | 0.02224 | <0.001 |
|  | worker | *in-vitro* queen | -1.38910(*) | 0.02793 | <0.001 |
|  |  | natural queen | -1.52392(*) | 0.02224 | <0.001 |
| WUT4 | *in-vitro* queen | natural queen | -0.00136 | 0.05105 | 1 |
|  |  | worker | .73240(*) | 0.04873 | <0.001 |
|  | natural queen | *in-vitro* queen | 0.00136 | 0.05105 | 1 |
|  |  | worker | .73376(*) | 0.03881 | <0.001 |
|  | worker | *in-vitro* queen | -.73240(*) | 0.04873 | <0.001 |
|  |  | natural queen | -.73376(*) | 0.03881 | <0.001 |
| TW5 | *in-vitro* queen | natural queen | -0.025 | 0.01332 | 0.154 |
|  |  | worker | .77892(*) | 0.01271 | <0.001 |
|  | natural queen | *in-vitro* queen | 0.025 | 0.01332 | 0.154 |
|  |  | worker | .80392(*) | 0.01012 | <0.001 |
|  | worker | *in-vitro* queen | -.77892(*) | 0.01271 | <0.001 |
|  |  | natural queen | -.80392(*) | 0.01012 | <0.001 |
| TL5 | *in-vitro* queen | natural queen | 0.00136 | 0.02449 | 0.998 |
|  |  | worker | 1.71543(*) | 0.02338 | <0.001 |
|  | natural queen | *in-vitro* queen | -0.00136 | 0.02449 | 0.998 |
|  |  | worker | 1.71407(*) | 0.01861 | <0.001 |
|  | worker | *in-vitro* queen | -1.71543(*) | 0.02338 | <0.001 |
|  |  | natural queen | -1.71407(*) | 0.01861 | <0.001 |
| WUT5 | *in-vitro* queen | natural queen | -0.01682 | 0.0234 | 0.753 |
|  |  | worker | .74023(*) | 0.02234 | <0.001 |
|  | natural queen | *in-vitro* queen | 0.01682 | 0.0234 | 0.753 |
|  |  | worker | .75705(*) | 0.01779 | <0.001 |
|  | worker | *in-vitro* queen | -.74023(*) | 0.02234 | <0.001 |
|  |  | natural queen | -.75705(*) | 0.01779 | <0.001 |
| SW4 | in vitro queen | natural queen | -0.01182 | 0.00858 | 0.359 |
|  |  | worker | .44509(*) | 0.00819 | <0.001 |
|  | natural queen | *in-vitro* queen | 0.01182 | 0.00858 | 0.359 |
|  |  | worker | .45691(*) | 0.00652 | <0.001 |
|  | worker | *in-vitro* queen | -.44509(*) | 0.00819 | <0.001 |
|  |  | natural queen | -.45691(*) | 0.00652 | <0.001 |
| SL4 | *in-vitro* queen | natural queen | -0.00636 | 0.02413 | 0.962 |
|  |  | worker | 2.88370(*) | 0.02303 | <0.001 |
|  | natural queen | *in-vitro* queen | 0.00636 | 0.02413 | 0.962 |
|  |  | worker | 2.89007(*) | 0.01834 | <0.001 |
|  | worker | *in-vitro* queen | -2.88370(*) | 0.02303 | <0.001 |
|  |  | natural queen | -2.89007(*) | 0.01834 | <0.001 |
| WNS4 | *in-vitro* queen | natural queen | -0.002 | 0.00521 | 0.922 |
|  |  | worker | .39774(*) | 0.00498 | <0.001 |
|  | natural queen | *in-vitro* queen | 0.002 | 0.00521 | 0.922 |
|  |  | worker | .39974(*) | 0.00396 | <0.001 |
|  | worker | *in-vitro* queen | -.39774(*) | 0.00498 | <0.001 |
|  |  | natural queen | -.39974(*) | 0.00396 | <0.001 |
| SW5 | *in-vitro* queen | natural queen | -0.02782 | 0.01889 | 0.311 |
|  |  | worker | .85805(*) | 0.01803 | <0.001 |
|  | natural queen | *in-vitro* queen | 0.02782 | 0.01889 | 0.311 |
|  |  | worker | .88587(*) | 0.01436 | <0.001 |
|  | worker | *in-vitro* queen | -.85805(*) | 0.01803 | <0.001 |
|  |  | natural queen | -.88587(*) | 0.01436 | <0.001 |
| SL5 | *in-vitro* queen | natural queen | -0.01045 | 0.02656 | 0.918 |
|  |  | worker | .65885(*) | 0.02535 | <0.001 |
|  | natural queen | *in-vitro* queen | 0.01045 | 0.02656 | 0.918 |
|  |  | worker | .66930(*) | 0.02019 | <0.001 |
|  | worker | *in-vitro* queen | -.65885(*) | 0.02535 | <0.001 |
|  |  | natural queen | -.66930(*) | 0.02019 | <0.001 |
| WNS5 | *in-vitro* queen | natural queen | -0.00045 | 0.01215 | 0.999 |
|  |  | worker | .56116(*) | 0.0116 | <0.001 |
|  | natural queen | *in-vitro* queen | 0.00045 | 0.01215 | 0.999 |
|  |  | worker | .56161(*) | 0.00924 | <0.001 |
|  | worker | *in-vitro* queen | -.56116(*) | 0.0116 | <0.001 |
|  |  | natural queen | -.56161(*) | 0.00924 | <0.001 |
