# Supplementary figures and images for: External morphometric and microscopic analysis of the reproductive system in in- vitro reared stingless bee queens, Heterotrigona itama, and their mating frequency
Source: PLoS One. 2024 Sep 24;19(9):e0306085. doi: 10.1371/journal.pone.0306085 (PMC11421791; doi:10.1371/journal.pone.0306085)

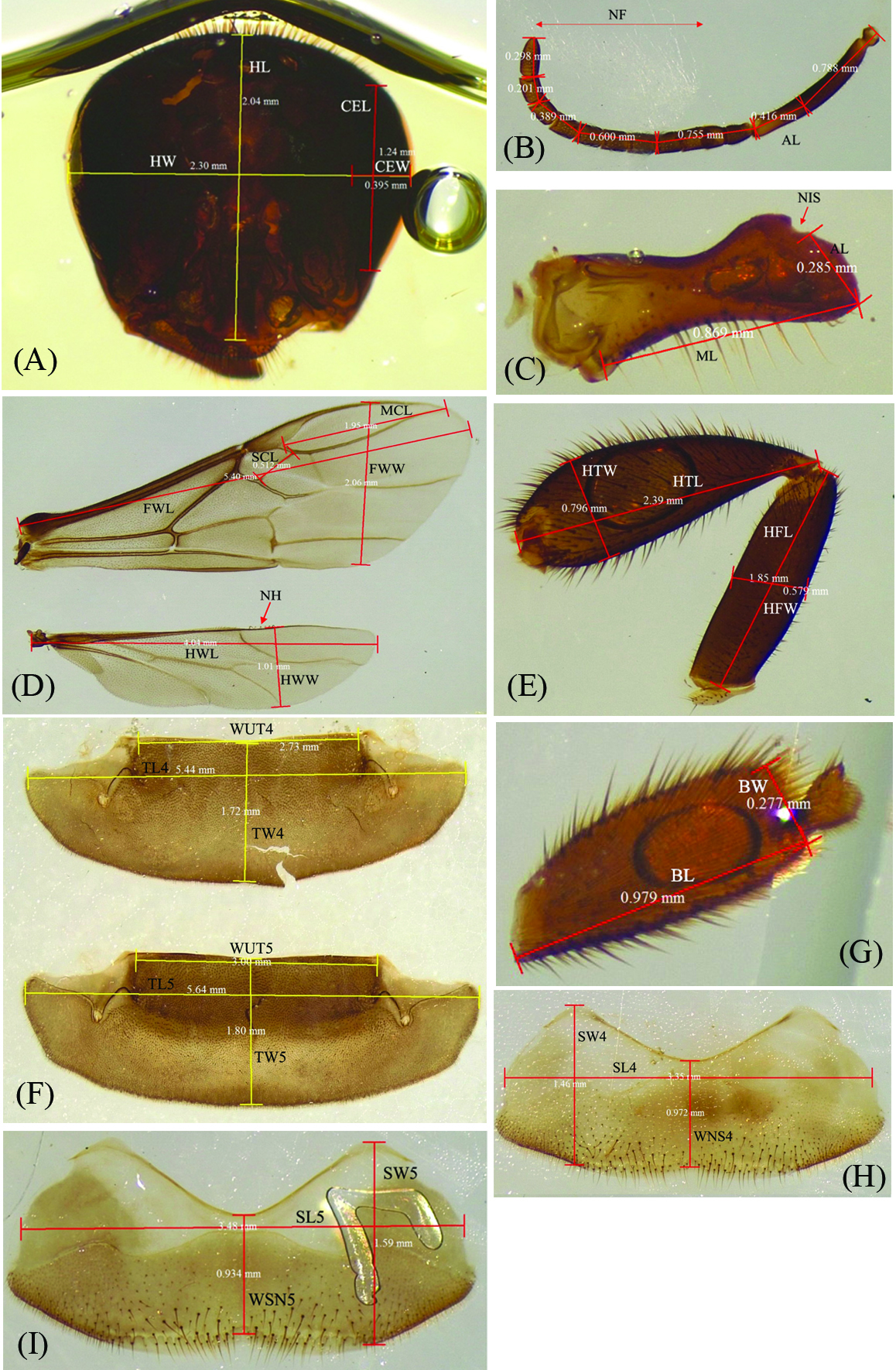

Supplement: S1 Fig — Thirty-four morphometric characters of in vitro queens, natural virgin queens, and young adult workers of Heterotrigona itama were examined, including (A) head, (B) antenna, (C) mandible, (D) forewing and hindwing, (E) hind femur and hind tibia, (F) 4th and 5th tergites, (G) hind basitarsus, (H) 4th sternite, and (I) 5th sternite. (TIF) [file pone.0306085.s004.tif]
